# Data Fusion to Develop a Driver Drowsiness Detection System with Robustness to Signal Loss

**DOI:** 10.3390/s140917832

**Published:** 2014-09-25

**Authors:** Sajjad Samiee, Shahram Azadi, Reza Kazemi, Ali Nahvi, Arno Eichberger

**Affiliations:** 1 Khaje Nasir Toosi University of Technology, Faculty of Mechanical Engineering, Tehran 19991-43344, Iran; E-Mails: Azadi@kntu.ac.ir (S.A.); Kazemi@kntu.ac.ir (R.K.); Nahvi@kntu.ac.ir (A.N.); 2 Graz University of Technology, Automotive Engineering Faculty, Inffeldgasse 11, Graz 8010, Austria; E-Mail: Arno.Eichberger@tugraz.at

**Keywords:** sensors data fusion, driver drowsiness detection, driver assistance system, eye tracking, vehicle lateral position, steering angle, vehicle safety, driving simulator

## Abstract

This study proposes a drowsiness detection approach based on the combination of several different detection methods, with robustness to the input signal loss. Hence, if one of the methods fails for any reason, the whole system continues to work properly. To choose correct combination of the available methods and to utilize the benefits of methods of different categories, an image processing-based technique as well as a method based on driver-vehicle interaction is used. In order to avoid driving distraction, any use of an intrusive method is prevented. A driving simulator is used to gather real data and then artificial neural networks are used in the structure of the designed system. Several tests were conducted on twelve volunteers while their sleeping situations during one day prior to the tests, were fully under control. Although the impact of the proposed system on the improvement of the detection accuracy is not remarkable, the results indicate the main advantages of the system are the reliability of the detections and robustness to the loss of the input signals. The high reliability of the drowsiness detection systems plays an important role to reduce drowsiness related road accidents and their associated costs.

## 1. Introduction

Driver drowsiness is a very important cause of many car accidents all around the world [1]. There are many casualties every year as a result of drowsy driving. In a study conducted in 2006, 109 vehicles equipped with cameras, sensors and data accusation tools, were monitored for a period of 12 to 13 months. The results showed that the drowsy drivers were involved in 22 percent of all car accidents [2]. Another study performed on truck drivers revealed that almost 20 percent of all accidents are caused by the sleepy drivers [3].

Loss of the consciousness due to the sleepiness causes some changes in human's body and activities [4]. These symptoms and parameters enable us to successfully measure the drowsiness level. Various methods of drowsiness detection can be divided into two general categories. The methods in the first group distinguish the level of the drowsiness based on the physiological changes in the body. Eye status, speech properties, time interval between two yawning, head position, sitting posture, heart rate, and brain signals are just a few examples of the methods in the first category. Drowsiness also results in some changes in the driving behavior and style. Methods in the second group estimate the driver's sleepiness level by tracking these changes. Steering angle, distance from the following vehicle, lateral position of the vehicle, longitudinal velocity, longitudinal acceleration, and lane departure are used in the method of the second category.

Driver's eye tracking is one of the most common methods of drowsiness detection applied in several studies [5–7]. PERCLOS (percentage of time the eyes are more than 80% closed) is known as the most effective parameter in the drowsiness detection [8].

Vitabile *et al.* used the PERCLOS parameter in their study. They evaluated the performance of their designed system in real driving conditions [9]. It was demonstrated in [10] that the frequency of the blinking as well as the eye vertical movement increases just before sleeping. They used these factors in their research to detect the drowsiness. Voice recognition and speech properties were used by [11]. The best performance of the system showed 81.6% accuracy, which was obtained using support vector machines. In another study done by Azim *et al.*, the driver yawning was used for sleepiness detection [12]. In this study, the Viola-Jones method was used for face recognition, which was previously introduced in [13]. In a very recent study, in 2014, Jo *et al.*, used feature-level fusion and user-specific classification to detect driver drowsiness. With the help of 2D Gaussian PDF, their system was able to model driver-specific blinking patterns for normal (non-drowsy) driving [14].

Different analyses were conducted on the driver drowsiness using the real data gathered from 10,300 drivers with the total driving distance of 1.23 million kilometer. The minimum error of 16.6% was obtained using the artificial neural networks in this research [15].

Mortazavi *et al.* showed that the sleepiness has a very large effect on the driver's control behavior over the steering wheel. It reduces the steering wheel angle variation and also results in large and sudden changes in the steering angle to correct the vehicle path [16]. In another research, the lateral position of the vehicle was used to detect the driver drowsiness. Data processing was conducted on the real data, gathered from the real driving situations. The results represented that the drowsy drivers often prefer to drive in middle lane of the road. More safety margin and fewer situations that need driver's reaction may be good justifications for this finding [17]. Use of the brain signals (EEG) is a very reliable method for detecting the level of the drowsiness [18–20]. In a research conducted in 2012, neural and Neuro-fuzzy systems, such as SVR (Support Vector Regression), MLPNN (Multi-Layer Perceptron Neural Network), RBFNN (Radial Basis Function Neural Network), and SONFIN (Self-Constructing Neural Fuzzy Inference Network) were used as a decision-making unit using the EEG signals [21]. Using wavelet analysis of heart rate variability and a support vector machine classifier was the main subject of the study done by Gang Li and Wan-Young Chung in 2013. They determined that a better real-time driver drowsiness detection system can be developed by using wavelet-based feature [22].

Borghini *et al.* performed a very comprehensive review about the neurophysiological signals in car drivers for the assessment of mental workload, fatigue and drowsiness [23] In another study, a non-intrusive approach for monitoring driver drowsiness using the fusion of several optimized indicators based on driver physical and driving performance measures was applied. In this research, Daza *et al.* proposed a new method for ground-truth generation based on a supervised Karolinska Sleepiness Scale (KSS) [24].

In many of the previous studies, the drowsiness detection system uses only one method to detect driver sleepiness. Various factors may cause such a system to easily fail and stop working correctly. Different methods and their possible corresponding errors are listed in the Table 1.

In the present research, the developed detection system does not only rely on one method. It uses a combination of the different methods to obtain the final result. Hence, if one of the methods fails for any reason, the whole system will continue to work correctly. While system accuracy is slightly reduced in such a circumstance, the detection system will be more robust and, hence, more reliable. To choose a correct combination from different available methods and in order to utilize the benefits of the methods of the different categories, a method based on the image processing as well as two methods based on the driver-vehicle interaction are selected. Additionally, to avoid driving distraction and reduce driver's tendency to use the system, no intrusive method is used.

## 2. Test Method

In this section, the procedure of conducting driving tests and collecting the required data are described.

### 2.1. Volunteers

Total number of 12 volunteers participated in the tests. To control two parameters of age and sex, all the drivers were selected among the male participants, aged between 21 and 28 years. No subject was addicted to drugs or alcohol and all of them had a valid driving license for at least two years and the experience of driving on intercity highways. All the drivers were checked to have a regular sleep pattern and without any sleep disorder.

### 2.2. Driving Simulator

**Figure 1.**
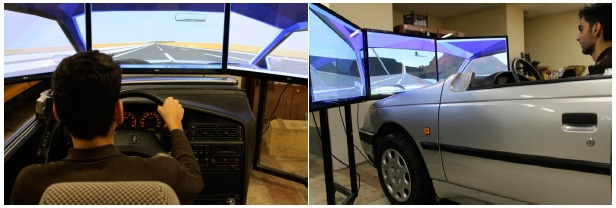
Driving simulator used to gather data.

A driving simulator, which was designed and constructed in the mechanical engineering faculty at Khaje Nasir Toosi University of Technology, used to gather the real data. Figure 1 shows two views of the driving simulator.

It is a fixed-base driving simulator with the active force feedback steering system and automatic transmission. Brake and gas pedals are equipped with the force and position sensors, respectively. The visualization system of the simulator uses three flat monitors and a sound system produces ambient and engine sounds. All communications and signal transmissions are carried out in real-time. The performance of the driving simulator has been already checked and validated [25].

### 2.3. Test Conditions

All tests were conducted in the early morning between 1 and 6 a.m. In order to create a steady environment and increase driver's willingness to sleep, tests were carried out in a quiet and dark room. The duration of the tests varied from one person to another and typically was in the range of 2 to 3 h.

### 2.4. Data Record

All required data were recorded in real-time with a sampling rate of 100 Hz. In addition to the vehicle dynamics, the driver's brain signals, as well as the data of the driver's face (front view), body (side view) and virtual driving path (driver's view) were recorded. Table 2 shows the complete list of all vehicle dynamics data recorded during the tests.

**Figure 2.**
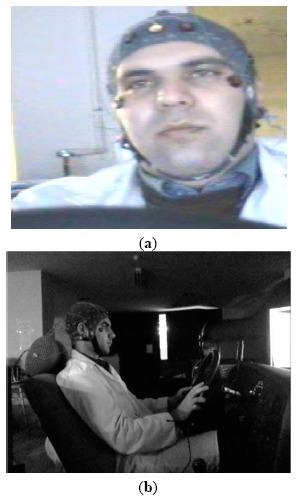
Images recorded by: (**a**) infrared camera and (**b**) low-lux camera in a dark room.

An infrared camera and a high-resolution low-lux camera were used to capture the driver's face and body, respectively. Figure 2 shows an example of the images recorded by each camera during the test conditions, discussed earlier.

### 2.5. Driving Path

**Figure 3.**
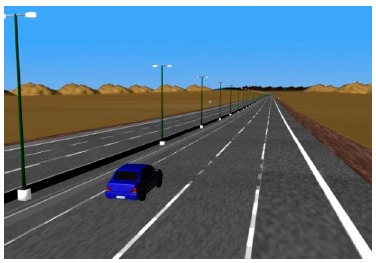
A view of the designed path.

Driving path was a looped trajectory with the initial length of 81 km and no intersections. The path included two different segments: a three-lane highway with the length of 52 km (beginning section) and a two-lane 29 km long mountain road (final section). Providing a very steady driving path is the main purpose in the road design procedure. Figure 3 shows a view of the designed path.

### 2.6. Scenarios

Participants were asked to drive in the fast-speed lane and avoid lateral deviations and lane departures during the test. They were also asked not to violate speed limitations and traffic signs. The participants were not allowed to stop the car in any case and were required to continue driving even though they feel sleepy.

## 3. Test Protocol

Before starting the tests, several tasks had to be done. These tasks are mentioned below:

- Each volunteer had to fill out a very comprehensive questionnaire, divided into different sections including demographic characteristics, medical history form, Epworth sleepiness scale questionnaire, and aggression questionnaire.
- All of the participants had to learn how to use the driving simulator. This is necessary to allow the participants to work with it easily and feel comfortable while driving (increasing sense of immersion).
- In order to familiarize the participants with the test environment and scenarios, each driver had to do some driving maneuvers in the driving simulator (duration of each pretest was approximately 20 min).
- EEG sensors had to be mounted on the participant's head.

Having confirmed all of the aforementioned tasks, each driver was allowed to start the real test. During the test, the driver was asked to rate his alertness based on the Karolinska sleepiness scale (KSS) in a specific time intervals. According to the test scenario, described earlier, each participant had to keep in the fast speed lane without the permission to stop the car. The tests were continued until a car collision happened due to the driver drowsiness. Drivers were asked to inform the operator in case of any dizziness, sickness, or headache during the test. All tests were carried out on 12 drivers in nine days with an approximate total driving time of 1650 min.

## 4. Drowsiness Scale

In many studies, the Karolinska Sleepiness Scale (KSS) has been used to evaluate the driver's level of drowsiness. The KSS is a self-reporting method in which the driver is asked every 15 min to give a number between 1 and 9 of his level of drowsiness (1 indicates completely aware and 9 denotes a very sleepy condition) [26]. Despite common usages of the KSS, this method has two limitations. Since the drowsiness is measured in 15 min intervals, the method cannot track the driver drowsiness continuously. Additionally, it is based on the self-assessment while based on our experiments the drivers do not often have an accurate sense about their level of drowsiness. To reduce these negative effects, two changes were made in the procedure. First, the time intervals between each measurement were reduced to 10 min. Then, in addition to the driver, two trained operators were asked to record the driver's drowsiness level based on the driving behavior and video of his face. The average value of these three numbers is assumed to be the real level of drowsiness. Finally, the data related to KSS ≥ 7 were considered as the drowsy driver while the data corresponding to KSS ≤ 5 were associated to an aware driver.

## 5. Input Signals

The input signals to the detection system are selected in such a way that loss of a signal does not jeopardize system performance. The input signals have been selected with care utilizing the signal processing techniques and driver-vehicle interaction techniques. Hence, the designed system takes the advantages of both methods. Moreover, to avoid driving distraction and reduce driver's tendency to use the system, no intrusive method is used. Finally, the vehicle's lateral position analysis, steering angle variation analysis, and driver's blinking pattern analysis were selected from the available methods.

### 5.1. Analysis of the Lateral Position of the Vehicle

**Figure 4.**
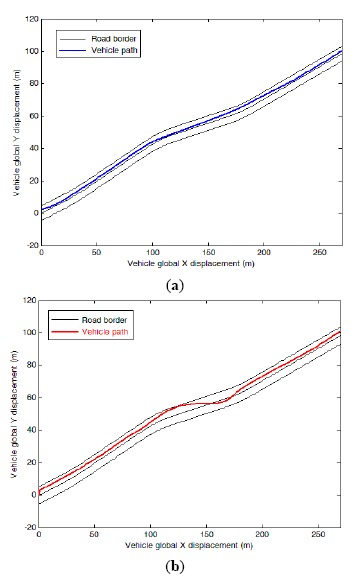
Vehicle path sample for a 20 s period and speed of 60 km/h. (**a**) Alert and (**b**) drowsy driver.

To analyze the lateral position of the vehicle, the lateral distance of the vehicle from the road center line has been employed. The lateral positions on the left side of the center line were assumed to be positive while the lateral position on the right side of the center line considered as negative values. Two sample signals obtained by this method for drowsy and conscious driver are shown in Figure 4. Based on this figure, the conscious drivers adjust the vehicle's path continuously and try to keep at the middle of the speed lane. On the other hand, the drowsy drivers are unable to keep the vehicle at the middle of the path and adjust the vehicle's position by applying large changes in the steer angle, only when the vehicle has been drifted into the road side. Hence, on an average basis the distance of the vehicle from the middle of the path is higher for the drowsy drivers. This fact serves as the basis for detection of the drowsy drivers in this study.

**Figure 5.**
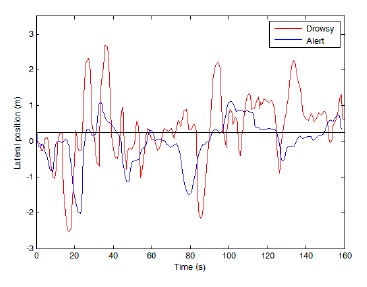
Vehicle lateral position within nine samples.

In Figure 5, the lateral position of the vehicle *vs.* time is demonstrated to compare a drowsy driver behavior with an alert driver within a longer sample of time. It can be seen that the lateral deviation in a conscious driver as well as maximum amount of lateral deviation is less than in a drowsy driver. Finally, the total amount of absolute lateral variations, as well as the mean squared error of lateral position was used as input signals.

### 5.2. Steering Angle

**Figure 6.**
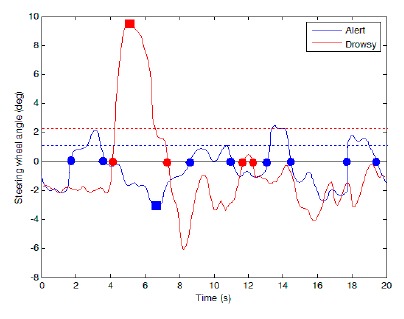
A sample of steering angle variations in a 20 s period.

During the analysis of the steering angle, three different features, including the number of zero-crossings, maximum value of the absolute steer angle, and average absolute steer angle have been used to distinguish the drowsy driver from the conscious one. Figure 6 shows an example of the variations in the steer angle for the drowsy and conscious drivers.

An alert driver continuously corrects the steer angle to keep the vehicle within the desirable path, as shown in Figure 6. Hence, compared to a drowsy driver, the number of the steer angle's zero-crossings for a conscious driver (marked by circles) is higher. On the other hand, maximum value of the absolute steer angle (marked by square), and average absolute steering angle (dashed line) associated with a drowsy driver is higher compared to a conscious driver. This due to the fact that drowsy driver find the vehicle deviation from the main path with more delay which requires big sudden changes in the steer angle as the vehicle drifts into either side of the road. In the analysis, the effect of road curves on the steering angle has been removed.

### 5.3. Driver's Blinking Status

**Figure 7.**
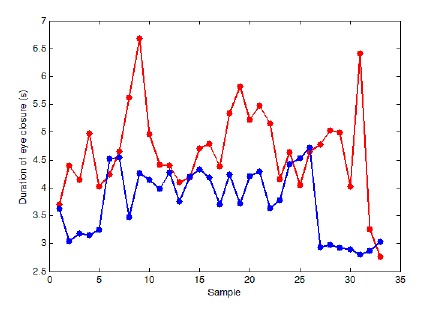
Duration of eye closure for 33 different time series.

To analyze driver's eye status, the duration that eyes are shut during a specified time interval is used. Usually, in the drowsy status, the frequency of the driver's eye blinking increases while the speed of the opening and closing of the eyelid decreases. In Figure 7 the marked points show the total duration that driver's eyes were closed for a 20 s period. This figure shows the status for a drowsy (red line) and a conscious (blue line) driver.

## 6. System Structure and Methodology Design

**Figure 8.**
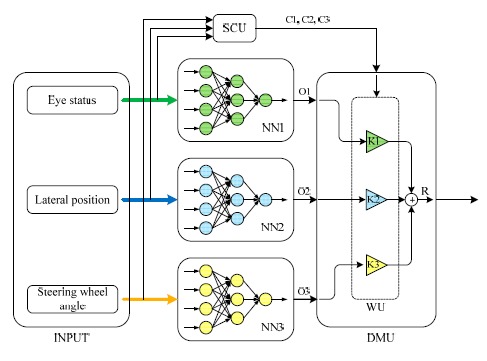
The structure of the proposed drowsiness detection system.

Neural networks are used for data processing in this study. Gathered information by each of three methods is first processed using a specific multi-layer perceptron (MLP). Hence, each network is able to distinguish the driver's level of drowsiness, independently. Then, a decision making unit (DMU) is used to measure the driver's drowsiness level considering the output of all neural networks and based on a simple algorithm which will be discussed in the following. The system structure is shown in Figure 8.

As demonstrated in Figure 8, the DMU multiplies the output of each MLP neural network by a number. Variables *k*_1_, *k*_2_, and *k*_3_, which are the gains of the corresponding drowsiness detection method in the final output will be set in WU (weighting unit). These gains are defined based on the accuracy of the methods in drowsiness detection; the more accurate methods would have greater impact in the final result. The algorithm that employed in the DMU can be mathematically summarized as follows:

(1) $$C_{1}k_{1}+C_{2}k_{2}+C_{3}k_{3}=1$$

(2) $$C_{1}k_{1}O_{1}+C_{2}k_{2}O_{2}+C_{3}k_{3}O_{3}=R$$

where, *O*_1_, *O*_2_ and *O*_3_ are the output of first, second, and third neural network, respectively. The output of each neural network is between 0 (indicating an aware driver) and 1 (indicating a drowsy driver). Moreover, *C*_1_, *C*_2_ and *C*_3_ are the output of Signal Check Unit and have a discreet value of 0 or 1, serving as an indicator for input signal availability. In the normal condition, were a detection method is working properly, the relevant *C_i_* should be 1. If for any reason (such as those mentioned in previous sections), a signal disruption happens in each of the applied methods, the corresponding neural network should not influence the decision-making procedure. Hence, in this situation the relevant value should be set to 0. Finally, if *R* ≥ 0.5 the driver is considered to be drowsy; otherwise he is assumed to be aware.

Using specific neural network for each method instead of one comprehensive neural network for the whole inputs may reduce the overall performance of the system. Feeding all data into one neural network can establish dependences between different inputs and, hence, result in better performance of the system. Although, using three different neural networks to detect the driver drowsiness will cause a small reduction in the system efficiency, it will bring a valuable opportunity to have a system, which is robust to signal loss and, hence, is more reliable.

A time series of 20 s was considered to calculate each input sample. All tests were carried out on 12 drivers with the total driving time of 1650 min. In order to familiarize the participants with the test environment and scenarios, each driver had to do some driving maneuvers as a pretest for a period of 20 min. The data gathered during pretests was not acceptable and must not be considered in the analysis. Thus, the total amount of 240 min was useless and was separated from the main data. In addition, 210 other min had to be removed because of system failure or interruptions during the tests, which caused drivers inattention. Finally, 1200 min of useful data was selected.

Applying a time series of 20 s to calculate a network input sample, the total amount of 3600 samples were obtained, of which, among them, 2194 samples were belonging to alert drivers while 1406 samples were classified as drowsy driver, based on KSS. To train the neural network, 70% of the data (1534 alert and 986 drowsy) was used as the training dataset, 15% (330 alert and 210 drowsy) are employed for the validation data, and 15% (330 alert and 210 drowsy) serve as the test data.

## 7. Experimental Results and Discussion

The MLP neural network was trained using back propagation learning algorithm. Different numbers of neurons in the hidden layer were tested and the best system architecture was found. First, each method was used alone for detection of a drowsy driver. Then, the test data were applied to the trained network and the outputs of the MLP were compared to the results obtained from KSS. The first neural network was trained using driver's eye blinking data. For the second neural network, the data of vehicle lateral position were used, and the third neural network was trained by the steer angle data. Table 3 presents the performance of each method in detection of the drowsiness.

In Table 3, TP (true positive) shows the performance of the system to correctly identify a drowsy driver, TN (true negative) represents the success rate of driver alertness detection, FP (false positive) demonstrates the failure percentage of system in classifying an alert driver as a drowsy one, and FN (false negative) illustrates the percentage of system error in identifying a sleepy driver as an conscious one.

According to the presented results in Table 3, detection based on analysis of the eye status produce more accurate results with an overall accuracy of 90.7%. The steer angle and lateral position methods result in 87.22% and 85.37% accuracy, respectively.

Next, the proposed technique in Section 6 was assessed. First, the data were fed to each neural network and then, by considering the outputs of the all networks simultaneously, the drowsiness of the driver was evaluated. Finally, the system output was compared to the results obtained from the KSS method. Moreover, the performance of the proposed technique in case of the input signals loss was investigated. Based on the presented results in Table 3, the eye analysis data (method number 1) plays a more decisive role in the final decision and, hence, attains a larger value for the constant (*K*_1_). For instance, in the situation that all signals are available (*C*_1_ = *C*_2_ = *C*_3_ = 1), considering Equation (1) and the result of each sole method in Table 3 the value for *K*_1_ to *K*_3_ can be calculated as the following:

(3) $$K_{i}=\frac{\text{Overal accuracy of method}\;i}{\sum_{j=1}^{3}\text{Overal accuracy of method}\;j}i=1,2,3$$

The first row of Table 4 corresponds to the case when all the input signals are properly received. It is seen that the proposed system is able to detect the drowsy driver with the accuracy of 94.63% which is a better results of the system compared to the performance of each sole method. Since the neural network is able to establish dependences between different features the system performance could be improved if we fed the inputs of all methods to a single neural network. However, then, the system could not overcome the signal loss and would become less reliable.

The second, third and fourth rows of Table 4 present the results obtained when the eye status signal, the lateral position signal, or the steer angle signal are lost, respectively.

Based on the presented results, although the overall accuracy degrades in comparison with the first row where all the inputs are available, the detection system does not fail and the proposed method is able to properly continue drowsiness detection.

## 8. Conclusions

This paper proposed a drowsiness detection system with the robustness to the loss of measured signals by fusion of data gathered from three different methods. Care was paid to the selection of the detection methods, such that in case of the any defect in one method, the remaining methods are able to detect the drowsiness, successfully. The experiments were conducted on 12 drivers by use of a driving simulator, under controlled sleep conditions. The neural networks were used in the structure of the proposed system for data fusion and detection of drowsiness. According to the obtained results, the following concluding remarks can be drawn:

- The designed system is able to properly work in case of the signal loss. In such circumstances, overall system performance was degraded by, at most, 6.85% and the accuracy of the detection in the worst case was 87.78%.
- The main advantages of the proposed system are the reliability of the detections and robustness to the loss of the input signals. In fact, impact of the proposed system on the improvement of the detection accuracy is, at most, 3.89%, which is not remarkable.
- The measurement data within a 20 s time interval are sufficient for the proposed system. In other words, the proposed system is able to detect the drowsiness status of the drivers by analyzing data captured within 20 s, resulting in lower computational burden, which makes it appropriate for real time purposes.

As discussed earlier, in the current system the classification is being done based on the value of *R*. Since the border between the two existing classes is not clearly separated, some errors might occur around the border region (*R* = 0.5). Thus, for future works, we aim to use more accurate system as decision-making unit. For instance, soft computing methods, such as work done in [27] will be used to obtain better results. Additionally, other nonintrusive methods will be examined to find a better combination of different drowsiness detection methods.

## Figures and Tables

**Table 1.** List of the different drowsiness detection methods and their possible cause of failure.

**Method**	**Restrictions**
Driver's face tracking (image processing methods)	1-Performance reduction in low ambient light
	2-Performance reduction while wearing glasses or having beard
	3-Tracking failure due to fast movements

Heart and brain signal processing	1-Intrusive method
	2-Reduce driver concentration
	3-Driver may forget to use (wear) the sensors

Driver reaction to a message	1-Intrusive method
	2-Not real-time

Lane departure warning	1-Unnecessary warnings
	2-System failure due to lack of clarity in road markings

Driver vehicle interaction/driving behavior	1-Closely dependent to driver's driving habits
	2-Changes by driver emotional state (anger, anxiety, sadness)

**Table 2.** Recorded vehicle dynamics data.

**Number**	**Variable**
1	Vehicle Longitudinal Position
2	Vehicle Lateral Position
3	Vehicle Longitudinal Velocity
4	Vehicle Lateral Velocity
5	Steering Wheel Torque
6	Steering Wheel Angle
7	Gas Pedal Position
8	Brake Pedal Force

**Table 3.** Performance of each method in drowsiness detection.

**Method**	**Driver Status**	**System Result**		**System Accuracy**				
	
		**Awake**	**Drowsy**	**TN**	**FP**	**FN**	**TP**	**Percentage of Overall Accuracy**
Blinking	Awake	303	27	91.72%	8.18%	10.95%	89.05%	90.74%
	Drowsy	23	187					

Lateral Position	Awake	295	35	89.39%	10.61%	20.95%	79.05%	85.37%
	Drowsy	44	166					

Steering Angle	Awake	292	38	88.48%	11.52%	14.77%	85.23%	87.22%
	Drowsy	31	179					

**Table 4.** Performance of the designed system in case of input signal loss.

**Signal Status**	**Constants in DM**			**Awareness Status**			**Percentage of Overall Accuracy**
	
	***K*_1_**	***K*_2_**	***K*_3_**		**Awake**	**Drowsy**	
*C*_1_ = *C*_2_ = *C*_3_ = 1	0.345	0.324	0.331	Awake	312	18	94.63%
				Drowsy	11	199	

*C*_1_ = 0, *C*_2_ = *C*_3_ = 1	0	0.495	0.505	Awake	289	41	87.78%
				Drowsy	25	185	

*C*_2_ = 0, *C*_1_ = *C*_3_ = 1	0.510	0	0.490	Awake	296	34	90.19%
				Drowsy	19	191	

*C*_3_ = 0, *C*_1_ = *C*_2_ = 1	0.515	0.485	0	Awake	292	38	89.44%
				Drowsy	19	191	
